# A Rare Case of Granular Cell Tumor in the Groin: Importance of Excision With Negative Margins

**DOI:** 10.7759/cureus.84637

**Published:** 2025-05-22

**Authors:** Hui Un Kim, Tyler Etwaroo, Darren Williams, Allen Tsai, Penelope Mashburn

**Affiliations:** 1 General Surgery, Western Reserve Health Education/NEOMED, Warren, USA; 2 General Surgery, American University of Antigua, Osbourn, ATG

**Keywords:** granular cell tumor, pathology, positive surgical margin, rare benign tumor, surgical-wide excision

## Abstract

Granular cell tumors (GCTs) are rare neoplasms of Schwann cell origin that typically arise in the head, neck, and tongue. We present a case of a 64-year-old female who presented with a left groin mass. Surgical excision was performed, and histopathological analysis confirmed a GCT without malignant features. However, the excised mass had positive margins, necessitating re-excision to minimize the risk of recurrence. Surgical excision with negative margins remains the primary treatment for GCTs. The case highlights the importance of recognizing GCTs in uncommon anatomical locations and the importance of complete surgical excision to prevent recurrence.

## Introduction

Granular cell tumors (GCTs) are rare benign tumors first described by Abrikossoff in 1926 [1]. They arise from Schwann cells and account for approximately 0.5% of all soft tissue tumors. GCTs are generally benign; however, less than 1% exhibit malignant features based on clinical and histological findings [1,2]. They are more prevalent in females between the ages of 30 and 50 [3]. The ethology of GCTs may be associated with abnormal RAS/MAPK signaling pathway, which can be seen in neurofibromatosis type 1, LEOPARD syndrome, and Noonan syndrome [1]. GCTs presenting as multiple lesions have been shown to be closely associated with these genetic syndromes [1].

GCTs may originate in any part of the body, most commonly in the head and neck (70%), tongue (30%), breasts (5-15%), and limbs [3]. Involvement of the groin is rare, with few reported cases. GCTs of the groin are often misdiagnosed as reactive lymphadenopathy or a lipoma due to their uncommon presentation [4,5]. The clinical and histopathological features of GCTs must be discussed more widely to ensure awareness that this neoplasm may appear in areas that are often overlooked in clinical practice, such as the groin. Surgical excision is reported to be the main treatment for GCTs [1,4]. Although benign GCTs have a good prognosis, complete surgical excision with negative margins is crucial to reduce the risk of recurrence and potential malignant transformation [1,2,5].

## Case presentation

A 64-year-old Caucasian female with a past medical history of diabetes type 2, chronic obstructive pulmonary disease, and coronary artery atherosclerosis presented with a left labial soft tissue mass at the labia for the past several years. She reported occasional pain but denied having recent unintended weight loss or any other symptoms. The patient had no personal history of tumors or cancers. She was an active smoker. Physical examination revealed a well-localized, non-tender, non-mobile mass on the left outer labia measuring approximately 4 × 2 centimeters (cm) with no overlying skin changes or erythema. No lymphadenopathy was noted. Differential diagnosis at that time was lipoma or cyst. Given the location and the size of the mass, the decision was made to perform surgical excision of the left labial mass without imaging. The specimen went down to the subcutaneous tissues and measured 4 × 2 × 1 cm, and was sent for pathology. The sample consisted of irregular pale yellow-ivory dense fibrous tissue.

**Figure 1:**
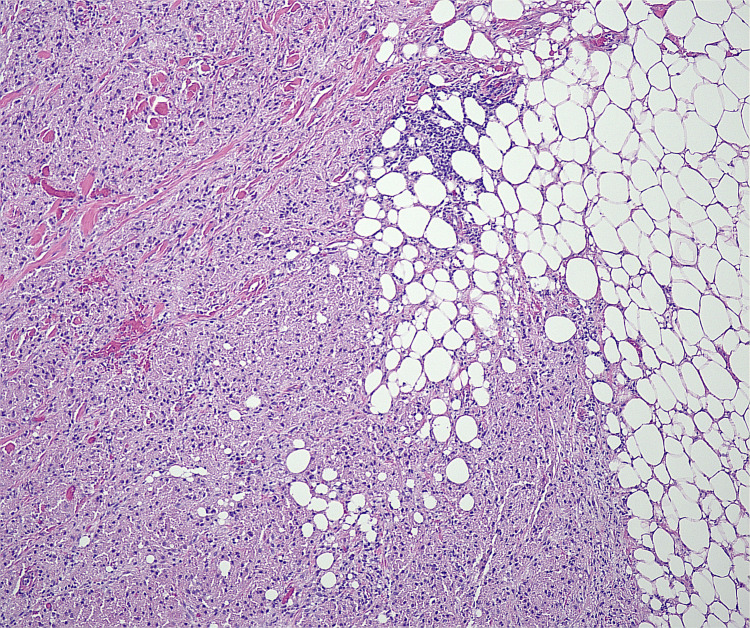
Low magnification of the granular cell tumor

**Figure 2:**
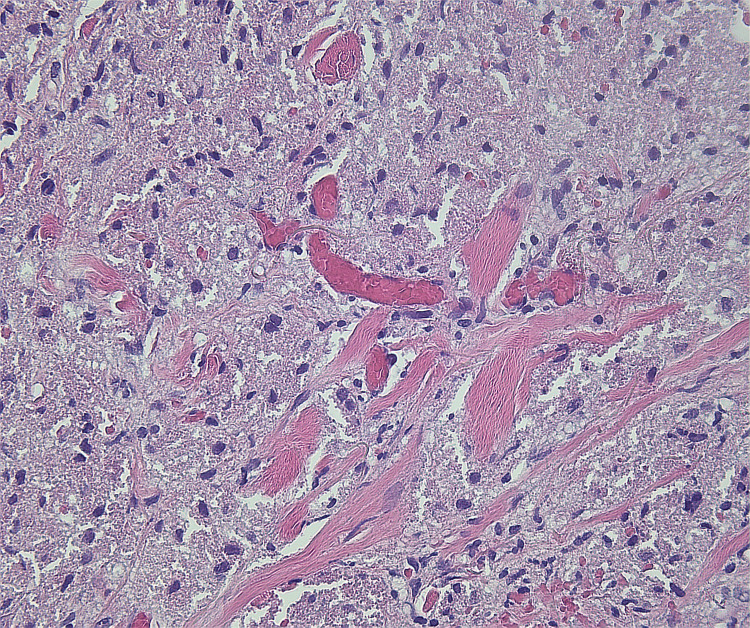
Higher magnification of the granular cell tumor

**Figure 3:**
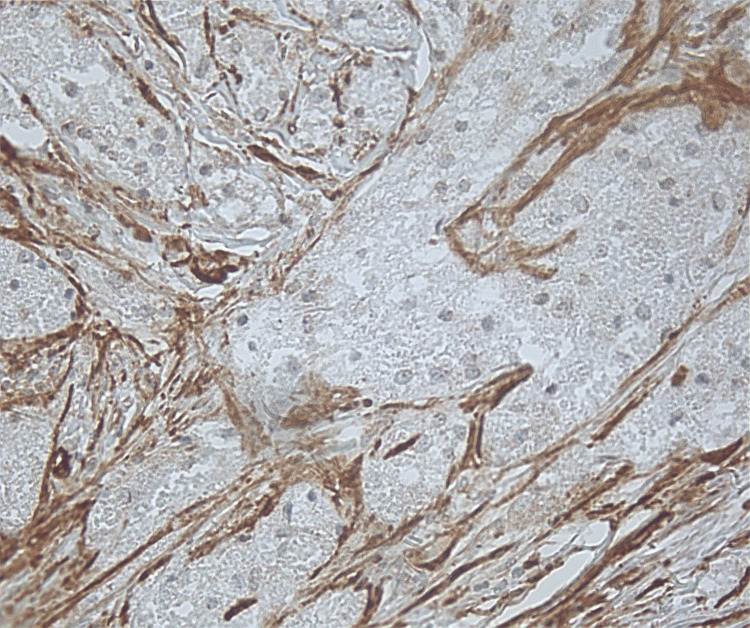
Smooth muscle actin is negative in tumor cells and positive in surrounding muscle, which the tumor is infiltrating into soft tissue

**Figure 4:**
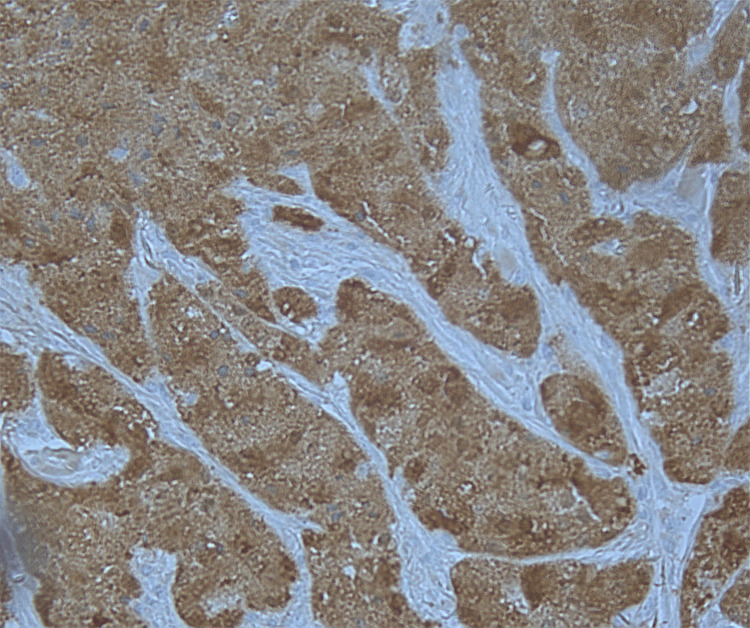
Immunohistochemical stain positive for S100

**Figure 5:**
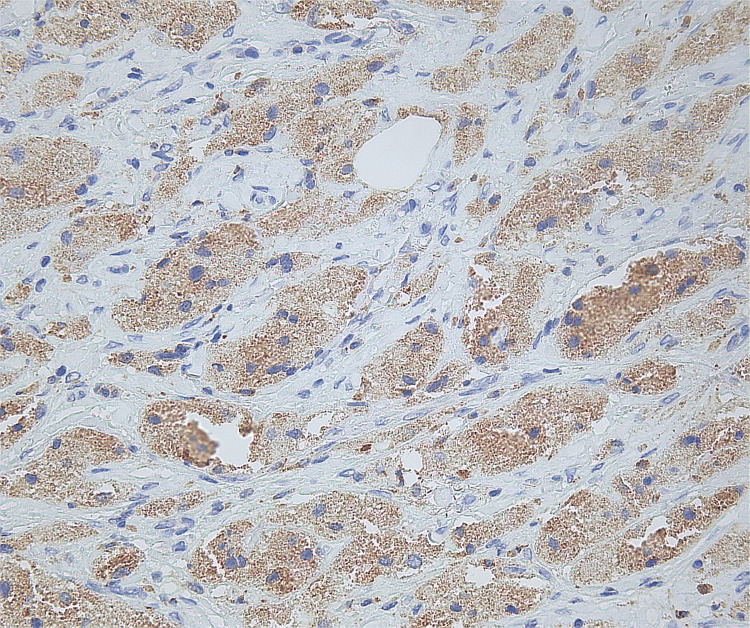
Immunohistochemical stain positive for CD68

**Figure 6:**
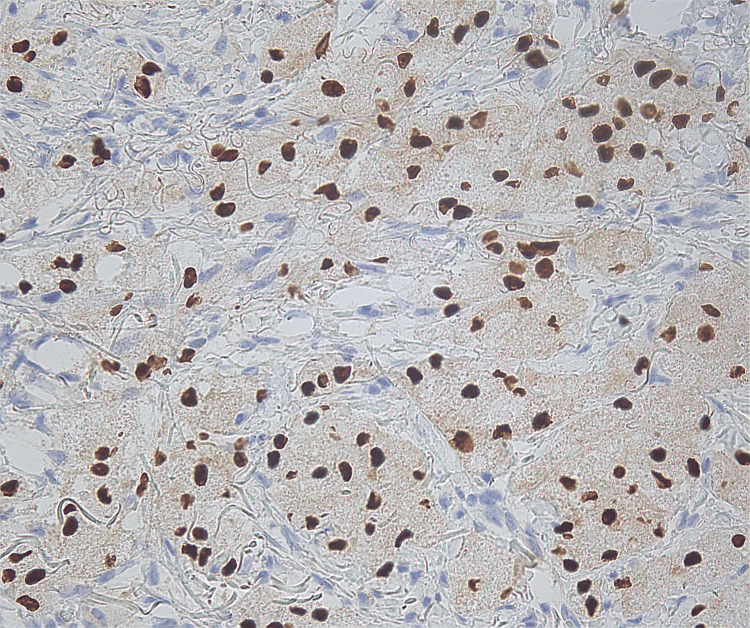
Immunohistochemical stain positive for SOX10

**Figure 7:**
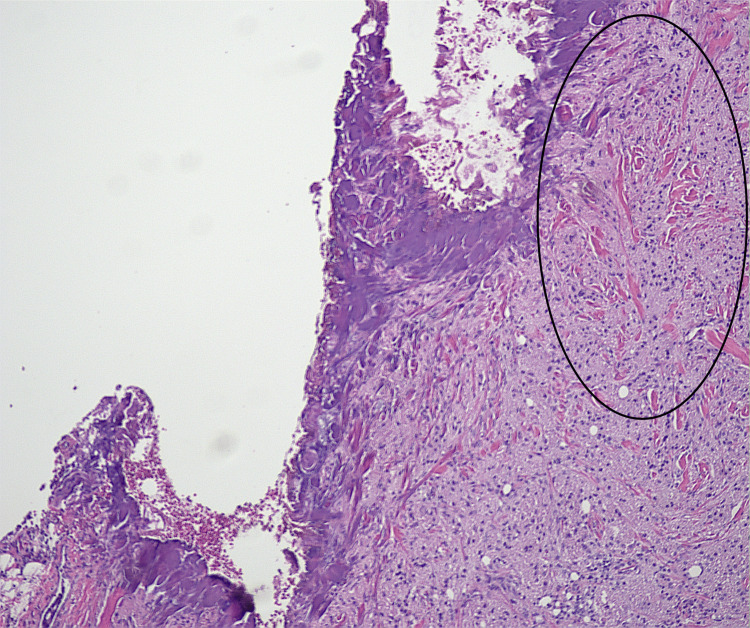
Positive margin on initial biopsy. Granular cell tumor is seen in the black circle. A similar pattern can be seen in figures 1 and 2.

Microscopic findings revealed proliferation of large round to oval cells with granular eosinophilic cytoplasm and small to intermediate-sized enlarged nuclei without significant nuclear atypia. No involvement of epidermis or ulceration, necrosis, increased number of mitoses, or significant atypia of nuclei was seen (Figures 1-2). Smooth muscle actin was negative in the tumor cells and positive in the surrounding muscle, showing infiltration of the tumor into the soft tissue (Figure 3). Tumor cells were positive for S100, CD68, and SOX10 (Figure 4-6), consistent with a diagnosis of GCT. The Ki-67 proliferation index immunostain was less than 5%. Pathology revealed positive margins (Figure 7), for which the patient underwent repeat wide local excision down to the healthy subcutaneous and fascia. Negative margins were achieved, with pathology findings consistent for benign GCT of the left labial mass. The patient was referred to oncology, however, general surgery follow-up was lost after confirmation of negative margins.

## Discussion

GCTs are uncommon neoplasms of Schwann cell origin, most frequently arising in the head, neck, and tongue [4]. Groin involvement is particularly rare and can pose a diagnostic challenge due to its clinical resemblance to more common benign lesions such as lipomas or reactive lymphadenopathy [4,5]. While the majority of GCTs are benign, the risk of malignant transformation and metastasis remains if left untreated [1,5]. Failure to obtain early imaging or biopsy may cause a delay in diagnosis, which may potentially increase the risk of incomplete excision and recurrence.

Imaging is not typically indicated prior to excisional biopsy of the small, benign-appearing nodules in the skin [1]. For this reason, no imaging was obtained for this patient preoperatively. Imaging is often indicated for tumors involving the breast, gastrointestinal tract, extremity soft tissue, or other atypical locations as they are indistinguishable from other benign or malignant lesions [1]. Differential diagnosis of GCTs in the mass in the groin can be lipoma, liposarcoma, lymphadenopathy, lymphoma, or metastatic tumor [1,4,5]. Definitive diagnosis of GCTs relies on histopathological evaluation, typically revealing polygonal cells with granular eosinophilic cytoplasm [3,6]. Fine-needle aspiration cytology has been proposed as an option to diagnose cutaneous GCTs [4]. Immunohistochemical staining plays a critical role in confirming Schwann cell origin, with markers such as S100, CD68, and SOX10 being positive in GCTs [7]. These features help distinguish GCTs from other soft tissue tumors and reactive lesions.

Fanburg-Smith et al. proposed histologic criteria to classify GCTs as malignant when three or more of the following features are present: necrosis, spindling, vesicular nuclei with large nucleoli, increased mitotic activity (>2 mitoses per 10 high-power fields), a high nuclear-to-cytoplasmic ratio, and pleomorphism [6,7]. Tumors exhibiting one or two of these characteristics are considered atypical [7]. Malignant GCTs are more likely to present as larger (>5 cm), subcutaneous masses, often located in the lower extremities, and may demonstrate clinical features such as rapid growth, ulceration, and local invasion. Common metastatic sites include the lungs, lymph nodes, and bones [3,7,8].

Surgical excision with negative margins remains the cornerstone of treatment. When excision is complete, the recurrence rate is approximately 2%; however, this can increase significantly to 21-50% in cases with positive margins [8,9]. In anatomically or cosmetically sensitive regions, Mohs micrographic surgery may be considered, although its application in GCTs remains limited and sparsely documented [8]. Malignant GCTs are known to respond poorly to chemotherapy and radiation therapy, further emphasizing the importance of early and complete surgical intervention [9].

Few cases of GCTs in the groin have been documented in the literature, and this case adds to the limited body of knowledge regarding such atypical presentations. Given the risk of recurrence, particularly in cases with positive or close margins, there is a need for close follow-up. Malignant GCTs can be diagnosed solely based on clinical findings such as rapid growth, large size (> 5 cm), ulceration, and invasion of local tissues [3,7-9]. Postoperative surveillance with imaging studies such as computed tomography (CT) scan or magnetic resonance imaging (MRI) may be an option. Preoperative imaging with CT scan and MRI would also be beneficial for screening for localization and even for lymphadenopathy and metastasis. Genetic testing for neurofibromatosis type 1, LEOPARD syndrome, PTEN hamartoma, and Noonan syndrome must be considered when GCTs present as multiple lesions [1]. However, a standardized surveillance protocol has not been established and may need to be considered in select patients with atypical features or incomplete resections.

## Conclusions

This is a rare presentation of GCT in the groin, an atypical anatomical location. Clinicians should be cautious when evaluating atypical soft tissue masses, even in uncommon locations such as the labia or groin, to ensure timely diagnosis and appropriate surgical management. Preoperative workup with imaging, such as CT or MRI, may be beneficial for localization and to screen for metastasis. Consideration for genetic testing should be made, especially if GCT presents as multiple lesions. Definitive diagnosis requires a biopsy, which can be obtained via fine-needle aspiration cytology or surgical excisional biopsy. Surgical treatment remains the main treatment for GCTs. Although GCTs are typically benign, incomplete excision increases the risk of recurrence. This highlights the importance of obtaining an accurate histological diagnosis and ensuring complete excision. Currently, there are no established postoperative surveillance guidelines dedicated to GCTs. More attention on postoperative surveillance is needed. Awareness of these rare presentations can help prevent misdiagnosis and reduce recurrence rates through timely and definitive surgical treatment.
